# Cervical Spondylotic Myelopathy Presenting as Peripheral Nerve Disease: A Case Report

**DOI:** 10.7759/cureus.47829

**Published:** 2023-10-27

**Authors:** Jonathan T Avon, Gavin J Scott, Sean M Bowling, Micah Jones

**Affiliations:** 1 Orthopedic Surgery, Corewell Health, Detroit, USA; 2 Orthopedics, Edward Via College of Osteopathic Medicine, Blacksburg, USA; 3 Orthopedic Surgery, WellSpan Health, York, USA; 4 Orthopedic Surgery, LewisGale Medical Center, Salem, USA

**Keywords:** carpal tunnel syndrome, hoffman sign, wartenberg sign, compressive myelopathy, degenerative cervical myelopathy

## Abstract

Cervical spondylotic myelopathy is the most common cause of spinal cord dysfunction in the elderly population. It is a degenerative disease that classically presents with fine motor dysfunction of the hands and gait instability. These symptoms can easily be masked by old age, complex medical history, and more benign diseases. We describe the case of a 67-year-old male who was referred to orthopedic surgery for bilateral hand numbness and weakness attributed to carpal tunnel syndrome (CTS). The patient had trouble ambulating, rhythmic clonus in his ankles, and a bilateral positive Hoffman sign resulting in a referral to neurosurgery for an emergent spinal cord decompression. To our knowledge, few case reports exist demonstrating how cervical myelopathy can mimic more benign peripheral nerve diseases such as CTS. We describe how difficult early recognition can be, as well as the importance of primary care doctors maintaining a high degree of suspicion for a disease that has nonspecific examination findings and can easily mimic more benign processes.

## Introduction

The carpal tunnel is located at the base of the palm and is bound by eight carpal bones and the transverse carpal ligament [1]. Residing within the carpal tunnel are nine flexor tendons as well as the median nerve [1]. Compression of the median nerve as it courses through the carpal tunnel into the wrist results in carpal tunnel syndrome (CTS). CTS is responsible for 90% of all nerve entrapment neuropathies and is prevalent in about 3.7% of the general population [2,3]. Clinically, patients with CTS present with decreased sensation in the median nerve distribution distal to the carpal tunnel. On examination, patients exhibit hand pain, numbness, and tingling of the thumb, index, middle, and radial side of the ring finger [4]. Nonsurgical management of CTS includes hand bracing, splinting of the wrist, oral steroids, nonsteroid anti-inflammatory drugs, and local injection of corticosteroids [2]. Surgical management of CTS consists of decompressing the carpal tunnel by division of the transverse carpal ligament [2].

More serious neurological diseases such as cervical spondylotic myelopathy (CSM) can be misdiagnosed because of more prevalent diseases such as CTS. CSM is the most common type of spinal cord dysfunction in patients older than 55 years and the most common cause of acquired spasticity later in life [5,6]. CSM is a spinal cord injury caused by compression of the spinal cord within the spinal canal [7]. Clinically, patients can present with both upper and lower neuron signs such as hyperreflexia, the Hoffmann sign, clonus, fasciculations, and the Babinski sign [8]. Patients may present with weakness and clumsiness in their upper extremities with associated difficulty performing everyday tasks [7]. Lower extremity weakness is also common among these patients and presents as a broad-based unsteady gait [7]. Severe CSM is a surgical emergency that requires decompressive surgery of the spinal cord [9].

## Case presentation

A 67-year-old male with a past medical history of hypertension, knee osteoarthritis, type 2 diabetes with peripheral neuropathy, and chronic pain from upper trunk/shoulder burns presented to the orthopedic clinic for evaluation of bilateral hand weakness. The patient’s surgical history was significant for bilateral total knee replacements as well as skin grafting in the upper extremities where the burns occurred. The patient noted that he had not had full range of motion in his right arm since the burns occurred. In the office, he complained that both hands had been in the form of contracture, impacting his ability to use either hand to its full capacity, with the right more severe than the left. He also reported intermittent swelling in both hands and had noticed that his hand issues started six months ago and continued to get progressively worse. He presented in a wheelchair but reported that he normally ambulates using a walker at home. The patient’s mobility had decreased drastically over the past six months. He had recently noticed difficulty with bowel and bladder control.

On arrival, the patient was afebrile with a temperature of 97.5°F, blood pressure of 112/72 mmHg, 100% oxygen saturation at room air, and body mass index of 27.12 kg/m^2^. On physical examination, the patient’s left hand showed a positive Wartenburg sign with no ability to actively abduct his small finger. He had weakness of the intrinsic muscles bilaterally and had a positive Hoffman sign bilaterally. The right hand had a contracted appearance, specifically the small and ring finger which could be corrected passively. He had five 5/5 strength regarding wrist extension and wrist flexion. The patient had 3+ reflexes bilaterally and had 0/5 flexion strength of the right elbow. Passive range of movement of the cervical spine showed no paresthesias, but he did have eight to nine beats of clonus bilaterally in his ankles, which was concerning for possible upper motor neuron lesion.

**Figure 1:**
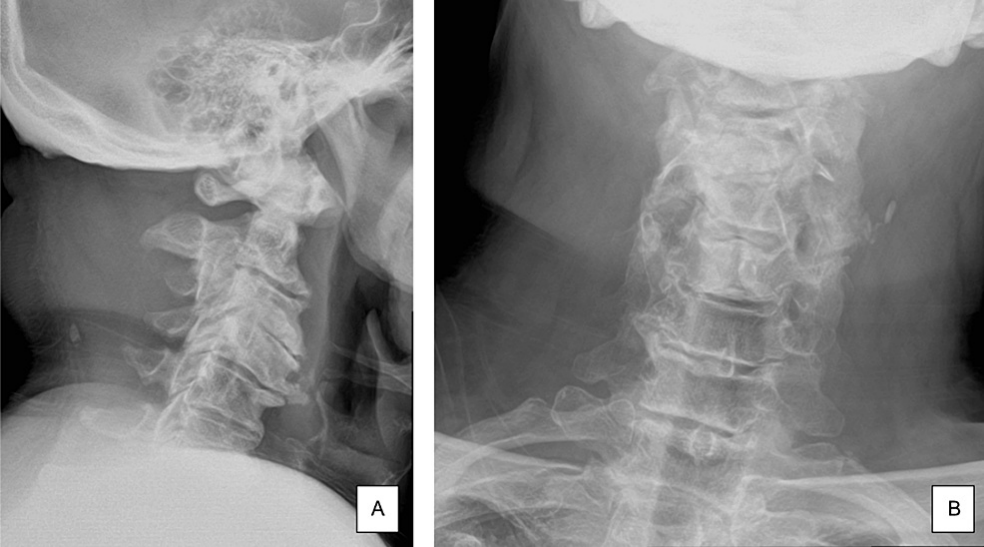
Radiographs of the cervical spine demonstrating severe degenerative changes at C3-C4. (A) Sagittal. (B) Anteroposterior.

**Figure 2:**
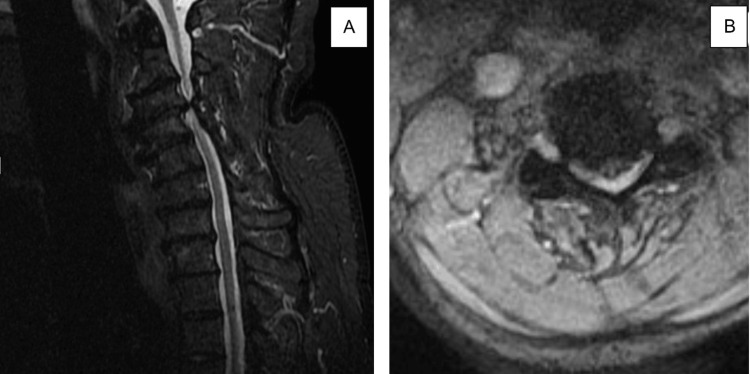
MRI of the cervical spine demonstrating severe cervical stenosis at C3-C4. (A) Sagittal. (B) Coronal.

The patient was sent for stat radiographs and an MRI of the cervical spine. Cervical radiographs (Figure 1) showed severe cervical spine degenerative disc disease with near-to-complete disk space height loss at C3-C4 with probable varying degrees of multilevel neural foraminal narrowing that could not be well characterized on radiographs. MRI (Figure 2) showed severe focal canal stenosis at C3-C4 with compression of the spinal cord and evidence for changes of compressive myelopathy. The radiologist noted that the patient could be set up for catastrophic cord injury with trivial trauma. Also noted on MRI was moderate anterior/posterior canal stenosis at C2-C3 with moderate-to-severe left and right foraminal stenosis. Moderate bilateral foraminal stenosis at C5-C6, C6-C7, and on the right at C7-T1 were noted as well.

An electromyography (EMG) nerve conduction study was also ordered to further rule out any peripheral nerve findings. Testing was limited to the patient’s left arm as the right arm had limited function. Left median and ulnar nerve studies showed normal motor latency with good amplitude and conduction velocity, ruling out concern for CTS. Increased tone in the muscles of the left upper extremity was noted on the EMG.

The patient was referred to neurosurgery where a cervical CT without contrast was ordered, revealing high-grade stenosis with compressive myelopathy at C3-C4 secondary to posterior ridging and ligamentum flavum hypertrophy. Neurosurgery performed decompression therapy with the fusion of the C3-C5 vertebrae. The patient’s leg strength improved almost immediately after surgery to 4/5 strength with improvement in hand coordination as well.

## Discussion

The objective of reporting this case is to demonstrate how CTS, a less severe disease process, can mask a more fatal disease such as cervical myelopathy. Although acute cases of CSM can be easily diagnosed, chronic cases are more likely to have an insidious onset that can mimic other diseases. This patient was referred to orthopedics for a nonemergent CTS consult which prolonged therapeutic intervention. This patient’s history and physical examination findings made an early diagnosis of cervical myelopathy difficult, especially in a primary care setting.

CTS is a well-documented peripheral neuropathy of the median nerve at the level of the wrist. Elevated pressure within the carpal tunnel produces ischemia of the median nerve, resulting in impaired nerve conduction, paresthesia, and pain [1]. The sensory distribution of the median nerve is the palmar aspect of the thumb, index finger, middle finger, and radial half of the ring finger [4]. Several provocative tests can be done to flare patients’ symptoms and help form a differential. The Phalen’s test is performed by placing the wrist into 90 degrees of flexion and asking if the patient has symptoms in the median nerve distribution [10]. Tinel’s sign is present if tapping over the volar surface of the wrist causes paresthesia down the patient’s hand [1]. The compression test is performed by applying force directly over the carpal tunnel for 30 seconds and asking if it reproduces the patients’ symptoms [1].

CSM can present much like CTS, especially early in the disease process. CSM can initially present with numbness and tingling in the extremities and progress to difficulty performing daily tasks [11]. Symptoms can also include neck, subscapular, and shoulder pain as well as sensory changes in the lower extremities [6]. The most common physical examination findings are motor weakness, hyperactive reflexes, clonus, and a spastic gait [6,11]. Babinski and Hoffman signs may be observed in degenerative CSM patients indicating upper motor neuron lesions. A Babinski sign can be visualized by stimulating the lateral aspect of the foot, causing the patient’s big toe to go into extension (dorsiflexion or upward movement) [12]. A Hoffman sign is an involuntary flexion movement of the thumb and or index finger when the examiner flicks the fingernail of the middle finger down [13].

With symptomatology overlapping, it is important to be able to tease out the differences between the two diseases before they progress. Cervical spondylosis precedes myelopathy in most cases making it crucial for primary care doctors to be able to differentiate cervical spondylosis from CTS. A study compared symptomatology in 44 patients who underwent surgery for CTS and 41 patients who underwent spinal surgery for cervical spondylosis. The authors found substantially higher rates of nocturnal paresthesia (84%), hand paresthesia that was aggravated by hand activity (82%), and hand pain (64%) in patients with CTS [14]. The incidences were only 10%, 7%, and 10%, respectively, in cervical spondylosis [14].

The challenges regarding this case can be partially attributed to this patient’s complex medical history. The patient’s mobility issues began shortly after his second knee replacement suggesting a possible complication of surgery. The patient’s trouble ambulating was not related to his recent surgery but to the compression of his spinal cord. This patient also had a history of bilateral upper extremity burns that resulted in bilateral skin grafts and upper extremity dysfunction. The patient’s medical background concealed the typical indicators of cervical myelopathy, such as a spastic gait and fine motor weakness in the hands, which are commonly observed in such cases. It is easy to see why his progressively worsening hand weakness was attributed to something less emergent such as CTS especially when 55-65% of carpal tunnel cases occur bilaterally [4].

## Conclusions

CTS is the most common peripheral nerve entrapment and gets diagnosed frequently in the primary care setting. Similarly, CSM is the most common spinal cord dysfunction that occurs in the elderly population. Symptomatology can easily overlap in the presence of a complex medical history and obscure physical examination findings. Physicians must rule out upper motor lesions in individuals who no longer ambulate and present with peripheral nerve entrapment symptoms.
